# Glyphosate-induced liver and kidney dysfunction, oxidative stress, immunosuppression in Nile tilapia, but ginger showed a protection role

**DOI:** 10.1007/s11259-022-09961-0

**Published:** 2022-06-30

**Authors:** Afaf D. Abdelmagid, Alshaimaa M. Said, Eman A. Abd El-Gawad, Sara A. Shalaby, Mahmoud A. O. Dawood

**Affiliations:** 1 Biochemistry Department, Faculty of Veterinary Medicine, Benha University, Banha, Egypt; 2 Aquatic Animal Diseases and Management Department, Faculty of Veterinary Medicine, Benha University, Banha, Egypt; 3 Department of Animal Production, Faculty of Agriculture, Kafrelsheikh University, Kafr el-Sheikh, Egypt; 4 The Center for Applied Research On the Environment and Sustainability, The American University in Cairo, Cairo, 11835 Egypt

**Keywords:** Blood metabolites, Herbicides, Medicinal herbs, Aquaculture nutrition, Immunity, Oxidative stress

## Abstract

The water-borne herbicides are involved in the toxicity of aquatic animals resulting in impaired health status and low productivity. Dietary medicinal herbs present a practical solution to relieve the impacts of herbicides toxicity on the performances of aquatic animals. Herein, we investigated the toxicity of commercial glyphosate-induced oxidative stress, immunosuppression, liver and kidney dysfunction, and the protective role of ginger or ginger nanoparticles in Nile tilapia. Fish were allocated into four groups: the first group presented the control without glyphosate toxicity and ginger feeding, the second group intoxicated with glyphosate at 0.6 mg/L and fed ginger free diet, the third group intoxicated with glyphosate and fed ginger at 2.5 g/kg, and the fourth group intoxicated with glyphosate and fed ginger nanoparticles at 2.5 g/kg. Fish were kept under the experimental conditions for four weeks, and the samples of blood and tissues were collected after 2 and 4 weeks. Markedly, fish exposed to glyphosate showed the highest ALT and AST activities, glucose and cortisol levels, and malondialdehyde levels (MDA) in gills and tissues. While fish in the control and fish intoxicated with glyphosate and fed ginger nanoparticles had the lowest ALT and AST activities, glucose and cortisol levels, and MDA levels after 2 and 4 weeks (*P* < 0.05). Fish fed dietary ginger had lower ALT and AST activities, glucose and cortisol levels, and MDA levels than the glyphosate intoxicated group after 2 and 4 weeks (*P* < 0.05). Interestingly, fish-fed ginger nanoparticles showed lower urea and creatinine levels and higher total protein, albumin, and globulin than the glyphosate intoxicated group (*P* < 0.05) and similar to the control (*P* > 0.05). Further, fish intoxicated with glyphosate and fed ginger nanoparticles had the highest GSH, lysozyme activity, and immunoglobulin levels after 2 and 4 weeks (*P* < 0.05). In conclusion, ginger nanoparticles are superior to the standard ginger form in enhancing the antioxidative and immune responses of Nile tilapia exposed to glyphosate.

## Introduction

There is a close relationship between aquaculture and agriculture activities where both are depending mainly on the drainage water for cultivation (Gewaily et al. 2021). Nevertheless, the heavy usage of herbicides and insecticides in agriculture to protect from herbals and insects is a limiting factor for the aquaculture sector (Bojarski and Witeska 2020). Usually, aquaculture activity depends on the reuse of the drainage water from the agriculture sector due to the limitation of water resources in some countries (Rossi et al. 2020). The waterborne derivatives of herbicides and pesticides negatively affect aquatic animals' health status and productivity (Dar et al. 2022; Naiel et al. 2020). Glyphosate is a very toxic herbicide involved in the protection against common herbs grown on crop farms and is known for its carcinogenic effects (Van Bruggen et al. 2018). Exposure to subchronic glyphosate led to oxidative stress, immunosuppression, inflammation, and apoptosis in various fish species (Ma et al. 2019; Mohapatra et al. 2021; Yalsuyi et al. 2021). The accumulation of herbicides, pesticides, and insecticides in the farming water affects the gills, skin, and intestines tissues of fish as the first mucosal lines directly contact the surrounding water (Banaee et al. 2020; Saha et al. 2021; Yang et al. 2020). More importantly, herbicide toxicity induces an imbalance in producing and removing reactive oxygen metabolites (ROS), leading to high lipid peroxidation and oxidative stress (Yang et al. 2020). Consequently, the toxic derivatives led to impairment in the entire body of fish's metabolic, physiological, and immunological conditions (Dawood et al. 2020a; Sutili et al. 2020).

Sustainable clean approaches are recently applied to ensure aquatic animals' well-being and high productivity under biotic and abiotic stressors (Paray et al. 2021). Nowadays, the focus is on applying eco-friendly functional additives in aquafeed to enhance aquatic animals' metabolic, physiological, and immunological responses (Elumalai et al. 2021). In this regard, medicinal herbs got close attention associated with their rich bioactive components and high functionality (Brum et al. 2018; Cardoso et al. 2021). The high pharmacological potential of medicinal herbs as natural antioxidative and anti-inflammation agents suggests using these additives to relieve the impacts of herbicides, insecticides, and pesticides on aquatic animals (Sinha et al. 2021; Yousefi et al. 2021). *Zingiber officinale* or ginger is a natural medicinal plant with high bioactive properties (Morvaridzadeh et al. 2020, 2021). The dietary inclusion of ginger led to marked enhancement in the growth performance, intestinal health, digestion capacity, antioxidative, and immune responses of several fish species (Chung et al. 2021b; Fazelan et al. 2020; Korni and Khalil 2017). Further, high resistance against bacterial infection was seen in infected aquatic animals due to the high antibacterial effect of ginger (Chung et al. 2021a; Korni et al. 2021). The production of ginger nanoparticles was also illustrated as an innovative approach for maximizing the functionality of ginger (Korni et al. 2021). In aquaculture, ginger nanoparticle usage resulted in high immunity and resistance to infection with pathogenic bacteria (Korni et al. 2021). Recently, it has been stated that ginger supplementation relieved the negative impacts of dimethoate exposure on the antioxidative response and histological features of gills, liver, and kidneys in Nile tilapia (*Oreochromis niloticus*) (Soror et al. 2021).

Nile tilapia is a freshwater fish species with high commercial value and can be cultured in the drainage water resulting from the agriculture sector (El-Sayed 2019). In Nile tilapia, sublethal toxicity of glyphosate induced negative histopathological impacts and inflammation features in the kidney (Hassan et al. 2022), gills (Jiraungkoorskul et al. 2003), liver (Abdelmagid et al. 2021), and spleen (Zheng et al. 2021) tissues. Further, immunosuppression (Abdelmagid et al. 2022), oxidative stress (Acar et al. 2021), and pro-inflammation responses were seen in Nile tilapia exposed to glyphosate herbicide. Therefore, it can be hypothesized that using ginger or its nanoparticles could relieve the negative impacts of glyphosate toxicity in Nile tilapia. This study aimed to evaluate the toxicity of glyphosate-induced oxidative stress, immunosuppression, liver and kidney dysfunction, and the protective role of ginger or ginger nanoparticles in Nile tilapia.

## Material and methods

### Ethical approval

All experimental techniques and fish care protocols used in the current study were followed by the Guidelines of Animal Care Use and were approved by the Institutional Animal Care Use Committee Research Ethics Board, Faculty of Veterinary Medicine, Benha University, Egypt.

### Ginger nanoparticles and test diets preparation

Ginger was obtained from the local market as a fine powder and stored in glass containers in the refrigerator until further use. Ginger nanoparticles were prepared by following Yadav et al. (2012) using a planetary ball mill (Retsch PM 400, Germany) at 550 rpm for 4 h till they reached size 100 nm. Commercial basal diets (30% crude protein, Uccam Food, Egypt) were crushed to a fine powder and split into three parts, with the first diet serving as the control. At a 2.5 g/kg diet dosage, the second and third diets were properly incorporated with ginger and ginger nanoparticles, respectively (Soror et al. 2021). Ginger and ginger nanoparticles were diluted in distilled water and mixed with a crushed diet to produce dough. The soft dough was re-pelleted using a meat mincer. The prepared pellets (2–3 mm) were kept at room temperature for 48 h to dry out, sealed in clean, dry plastic bags, and stored at 4 °C until needed.

### Fish and experimental procedure

A total of 250 healthy all males Nile tilapia (25.71 ± 0.5 g) have been obtained from a private fish farm at Kafr El-Sheikh Governorate, Egypt. Then, they were allocated in two fiberglass tanks (750 L capacity) provided with continuous aeration for two weeks acclimation period. During this period, fish were hand-fed daily with the control diet at a 3% body weight twice daily (8:00 and 16:00). Fish were stocked in twelve glass aquaria (89 × 30 × 29 cm) at 15 fish per aquarium. The aquaria present four groups in triplicates, and each aquarium is supplied with continuous aeration. The first group was fed a basal diet and kept in glyphosate-free water (control). The second group was intoxicated with glyphosate at 0.6 mg/L and fed the basal diet. The third group was intoxicated with glyphosate and fed ginger at 2.5 g/kg. The fourth group was intoxicated with glyphosate and fed ginger nanoparticles at 2.5 g/kg. The water from groups two to four was partially exchanged three times weekly, and the dose of glyphosate (Roundup 48%, Agrochem, Alwatneia Co., Alex., Egypt) was maintained within the required concentration (0.6 mg/L) in each aquarium. Meanwhile, the water was renewed with dechlorinated tap water in the control group. Glyphosate lethal concentration (LC50; 12 mg/L) was calculated earlier by Abdelmagid et al. (2021), and fish was exposed to 1/20 of LC50 (0.6 mg/L) following Abdelmagid et al. (2022). All fish have received the experimental diets twice daily at a feeding rate of 3% of the total body weight for four weeks under 12 h day:12 h night photoperiod regime. Water quality was maintained at 25 ± 1 °C, 5.1 ± 0.2 mg/L, 0.23 ± 0.07 mg/L, and 7.2 ± 0.2 for the temperature, dissolved oxygen, ammonia concentration, and pH, respectively. Water quality parameters (temperature, pH, and dissolved oxygen) were measured using Jenway, 370 pH meter, UK, and Crison OXI 45 P, EU, while total ammonia by following APHA (2017). The water exchange was done daily to eliminate fecal matter and uneaten food to maintain water quality parameters. The particle size distribution and zeta potential of ginger nanoparticles were determined using Zetasizer MS2000 (Malvern, United Kingdom). Additionally, the microstructure of ginger nanoparticles was examined by Ultraviolet spectrophotometer (V-750, Jasco Inc) by exposing the sample to visible light at rang 200-800 nm. The ginger nanoparticle morphology was observed under transmission electron microscopy (JEM2100, Japan) at an accelerating voltage of 200 K.V.

### Blood and tissue collection

Blood and tissue samples were collected from all treated groups after 2 weeks of exposure and at the end of the experiment (4 weeks). Fish were anesthetized with MS-222 (100 μg/mL), then blood samples from nine fish per treatment (3 fish/ replicate) were collected without anticoagulant. To separate serum, these samples were left in a slant position for 30 min and centrifuged at 3000 r.p.m for 15 min. The serum was collected and kept at -20 ˚C for immunological and biochemical assays.

For evaluation of antioxidant enzyme activity, liver and gills specimens were taken from nine fish per treatment (3 fish/ replicate), homogenized in 9 volumes of ice-cold 0.05 Mm potassium phosphate buffer saline PH 7.4 using a glass homogenizer. The homogenates were centrifuged at 6000 r.p.m for 15 min at 4˚C. The resultant supernatant was used to determine malondialdehyde (MDA) and reduced glutathione (GSH).

### Serum biochemical and immune assays

Serum glucose level was estimated according to the method reported by Trinder (1969). Cortisol was determined following the method described by Munro, and Lasley (1988), based on the competitive binding technique where cortisol competes with horseradish peroxidase (HRP)-labeled cortisol.

Serum alanine aminotransferase activity (ALT) was determined according to the method described by Huang et al. (2006). Aspartate aminotransferase activity (AST) was assayed according to Schumann et al. (2011). The urea level in serum was measured using commercial kits (Biomed, Germany) based on Levinson (1978) method. Creatinine in the serum was assayed following the protocol described by Moss et al. (1975). Serum total proteins and albumin were determined according to the method described by Doumas et al. (1981). The globulins concentration was calculated by subtracting the albumin values from the total protein values.

Serum lysozyme activity was determined according to the method described by Ellis (1990), depending on the lysis of *Micrococcus lysodeikticus* suspension. The activity was calculated from a standard curve prepared with lysozyme from chicken egg white. The concentration of serum total immunoglobulins was evaluated at 450 nm according to the method described by Siwicki (1993).

### Liver and gills antioxidant assay

The concentration of MDA in liver and gills tissues was determined based on the protocol of Esterbauer, and Cheeseman (1990)**,** which depends on the determination of thiobarbituric acid reactive substance content (TBARs) at 530 nm. Reduced GSH assay was based on GSH reaction with DTNB (5, 5`-dithiobis nitro benzoic acid) produces a yellow product of nitromercaptobenzoic acid that absorbs at 412 nm (Beutler 1963).

### Statistical analysis

All data were tested for normality and homogeneity by Shapiro–Wilk's and Levene's tests, respectively. Then, a one-way analysis of variance was used to determine the statistical significance of differences among groups, followed by Duncan's test as post hoc for making a multiple comparison using SPSS software (Version 25, SPSS Inc., Chicago, IL, USA). The values were expressed as the mean ± standard error. Mean values were considered significant at a *P*-value < 0.05 probability level.

## Results

### Characterization of ginger nanoparticles

**Fig. 1:**
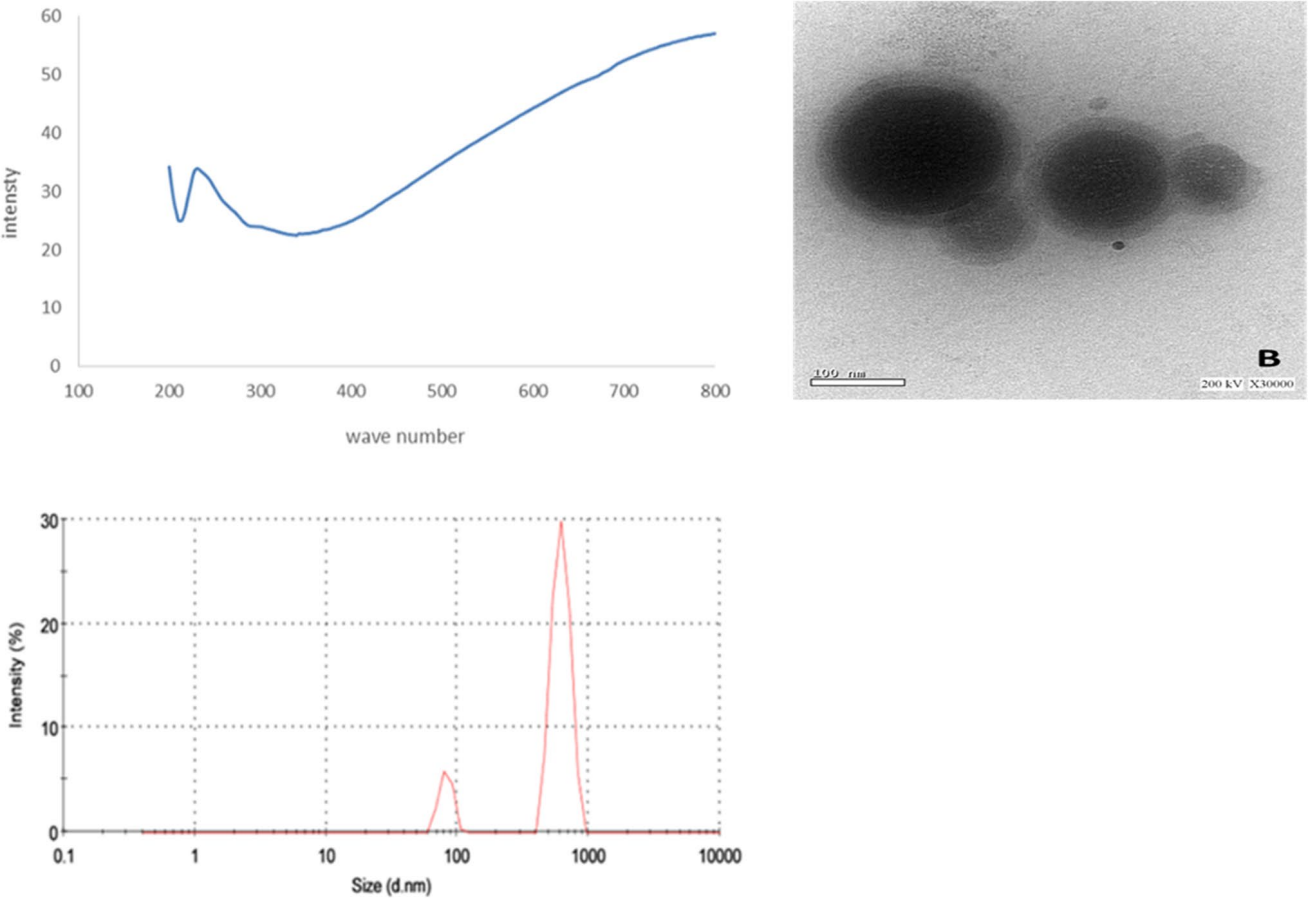
The ginger nanoparticles morphology was observed under transmission electron microscopy (JEM2100, Japan) at accelerating voltage of 200 K.V

As shown in Fig. 1, UV/VIS spectroscopy showed one peak of ginger nanoparticles at maximum absorbance 57.08 obtained at wavelength 340 nm. The size distribution of ginger nanoparticles analyzed by Zetasizer for size determination showed an average diameter of 906.9 d.nm. TEM image showed that ginger nanoparticles were circular in shape.

### Growth performance

**Fig. 2:**
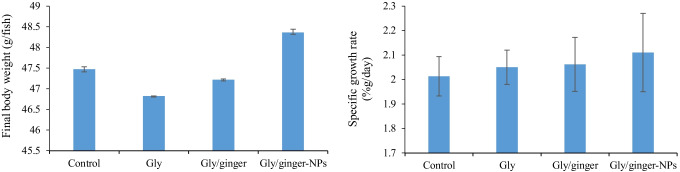
Final weight and specific growth rate of Nile tilapia fed ginger or ginger nanoparticles (NPs) after 4-week exposure to sublethal concentration of glyphosate (Gly). Specific growth rate (%g/day) = 100 × ((Ln final weight – Ln initial weight)/time in days)

The obtained results indicated that Nile tilapia-fed ginger or ginger nanoparticles after 4-week exposure to sublethal concentration of glyphosate had no significant differences in the case of final weight and specific growth rate (*P* > 0.05) (Fig. 2).

### Liver and kidney-related metabolites

Liver and kidney indicators in Nile tilapia fed ginger or its nanoparticles after 2- and 4-weeks exposure to glyphosate are shown in Table 1. Markedly, fish exposed to glyphosate showed the highest ALT and AST activities, while fish in the control group had the lowest ALT and AST activities after 2 and 4 weeks (*P* < 0.05). Fish fed dietary ginger had higher ALT, AST, urea, and creatinine levels than the control and lowered ALT, AST, urea, and creatinine levels than the glyphosate intoxicated group after 2 and 4 weeks (*P* < 0.05). Interestingly, fish-fed ginger nanoparticles showed lower ALT and AST activities than the glyphosate intoxicated group (*P* < 0.05) without significant differences from the control (*P* > 0.05). Further, urea and creatinine levels showed the lowest values meaningfully in fish fed the control diet without glyphosate toxicity, and fish fed ginger nanoparticles with glyphosate toxicity after 2 and 4 weeks (*P* < 0.05).

**Table 1:** Liver and kidney indicators in Nile tilapia fed ginger or its nanoparticles (ginger-NPs) after 2- and 4-weeks exposure to sub-lethal concentration of glyphosate (Gly)

Groups	ALT (U/L)		AST (U/L)		Urea (mg/dl)		Creatinine (mg/dL)	
	2 weeks	4 weeks	2 weeks	4 weeks	2 weeks	4 weeks	2 weeks	4 weeks
Control	9.93 ± 0.55c	11.03 ± 0.61c	16.55 ± 0.91c	19.86 ± 1.09c	12.33 ± 0.38c	10.28 ± 0.32c	0.40 ± 0.01c	0.42 ± 0.01c
Gly	53.25 ± 4.56a	61.53 ± 5.27a	65.08 ± 5.58a	80.47 ± 6.89a	41.26 ± 2.51a	45.73 ± 2.78a	2.62 ± 0.16a	3.01 ± 0.18a
Gly/ginger	26.58 ± 1.54b	29.53 ± 1.71b	44.30 ± 2.57b	53.16 ± 3.09b	16.57 ± 0.50b	13.82 ± 0.41b	1.54 ± 0.02b	1.07 ± 0.02b
Gly/ginger-NPs	13.44 ± 0.87bc	14.94 ± 0.97bc	22.41 ± 1.45bc	26.89 ± 1.74bc	12.39 ± 0.75c	10.33 ± 0.63c	0.90 ± 0.03bc	0.42 ± 0.03c

Values are presented as the mean ± S.E. (*n* = 3). Means within the same row carrying different letters are significantly different at *P* < 0.05 at each experimental time. *ALT* Alanine aminotransferase; *AST* Aspartate aminotransferase

### Blood proteins

The blood total protein, albumin, and globulin levels were meaningfully increased (*P* < 0.05) in the control group and severely reduced in fish exposed to glyphosate toxicity after 2 and 4 weeks (Table 2). No significant differences were seen between the control and fish intoxicated with glyphosate and fed ginger nanoparticles (*P* > 0.05). Moreover, fish fed dietary ginger and exposed to glyphosate had higher total protein, albumin, and globulin levels (*P* < 0.05) than the glyphosate intoxicated group without ginger feeding after 2 and 4 weeks.

**Table 2:** Blood total protein, albumin, and globulin in Nile tilapia fed ginger or its nanoparticles (ginger-NPs) after 2- and 4-weeks exposure to sub-lethal concentration of glyphosate (Gly)

Groups	Total protein (g/dL)		Albumin (g/dL)		Globulin (g/dL)	
	2 weeks	4 weeks	2 weeks	4 weeks	2 weeks	4 weeks
Control	5.74 ± 0.31a	5.85 ± 0.32a	3.39 ± 0.17a	3.61 ± 0.18a	2.35 ± 0.14a	2.24 ± 0.14a
Gly	1.86 ± 0.13c	1.60 ± 0.11c	1.34 ± 0.09c	1.07 ± 0.07c	0.52 ± 0.04c	0.53 ± 0.04c
Gly/ginger	2.93 ± 0.19b	2.98 ± 0.19b	1.77 ± 0.10ab	1.88 ± 0.10b	1.16 ± 0.09b	1.10 ± 0.09b
Gly/ginger-NPs	4.40 ± 0.18ab	4.49 ± 0.19ab	2.45 ± 0.15b	2.61 ± 0.16ab	1.96 ± 0.25ab	1.88 ± 0.25ab

Values are presented as the mean ± S.E. (*n* = 3). Means within the same row carrying different letters are significantly different at *P* < 0.05 at each experimental time

### Stress-related markers

Fish exposed to glyphosate showed the highest glucose and cortisol levels, while fish in the control group had the lowest glucose and cortisol levels after 2 and 4 weeks (*P* < 0.05) (Table 3). Fish fed dietary ginger had higher glucose and cortisol levels than the control and lower glucose and cortisol levels than the glyphosate intoxicated group after 2 and 4 weeks (*P* < 0.05). Interestingly, fish-fed ginger nanoparticles showed non-significant differences from the control after 2 and 4 weeks (*P* > 0.05).

**Table 3:** Stress biomarkers in Nile tilapia fed ginger or its nanoparticles (ginger-NPs) after 2- and 4-weeks exposure to sub-lethal concentration of glyphosate (Gly)

Groups	Glucose (mg/dL)		Cortisol (ng/mL)	
	2 weeks	4 weeks	2 weeks	4 weeks
Control	61.65 ± 1.91c	66.79 ± 2.07c	30.75 ± 0.43c	34.50 ± 0.87c
Gly	110.41 ± 6.71a	117.10 ± 7.11a	128.13 ± 2.39a	105.00 ± 2.89a
Gly/ginger	82.88 ± 2.49b	89.79 ± 2.69b	68.25 ± 1.01b	60.48 ± 0.85b
Gly/ginger-NPs	70.63 ± 2.41bc	67.11 ± 4.08c	52.50 ± 1.44bc	43.40 ± 0.81bc

Values are presented as the mean ± S.E. (*n* = 3). Means within the same row carrying different letters are significantly different at *P* < 0.05 at each experimental time

### Liver and gills antioxidant status

**Fig. 3:**
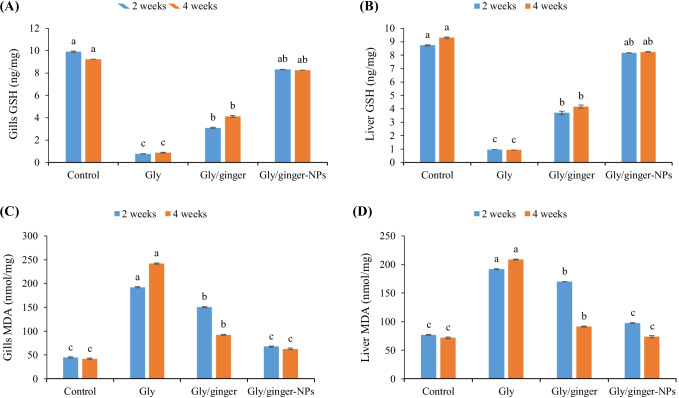
Antioxidant activity and lipid peroxidation level in Nile tilapia fed ginger or its nanoparticles (ginger-NPs) after 2- and 4-weeks exposure to sub-lethal concentration of glyphosate (Gly). Bars present means ± S.E. (*n* = 3) with different letters, differ significantly (*P* < 0.05). Reduced glutathione (GSH) and malondialdehyde (MDA) level

In the gills and liver tissues, fish exposed to glyphosate showed the lowest glutathione (GSH) levels (Fig. 3A and B), while fish in the control group and fish intoxicated with glyphosate and fed ginger nanoparticles had the highest GSH after 2 and 4 weeks (*P* < 0.05). Further, fish-fed dietary ginger had higher GSH than the glyphosate intoxicated group after 2 and 4 weeks (*P* < 0.05). Interestingly, fish-fed ginger nanoparticles showed non-significant differences from the control after 2 and 4 weeks (*P* > 0.05).

In the gills and liver tissues, fish exposed to glyphosate showed the highest malondialdehyde (MDA) levels (Fig. 3C and D), while fish in the control group and fish intoxicated with glyphosate and fed ginger nanoparticles had the lowest MDA levels after 2 and 4 weeks (*P* < 0.05). Further, fish-fed dietary ginger had a lower MDA level than the glyphosate intoxicated group after 2 and 4 weeks (*P* < 0.05).

### Immune response

**Fig. 4:**
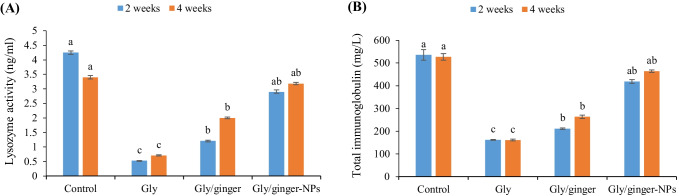
Lysozyme activity and immunoglobulin level in Nile tilapia fed ginger or its nanoparticles (ginger-NPs) after 2- and 4-weeks exposure to sub-lethal concentration of glyphosate (Gly). Bars present means ± S.E. (*n* = 3) with different letters, differ significantly (*P* < 0.05)

Markedly, fish exposed to glyphosate showed the lysozyme activity (Fig. 4A) and total immunoglobulin level (Fig. 4B), while fish in the control group and fish intoxicated with glyphosate and fed ginger nanoparticles had the highest lysozyme activity and total immunoglobulin level (*P* < 0.05). Further, fish-fed dietary ginger had higher lysozyme activity and total immunoglobulin levels than the glyphosate intoxicated group after 2 and 4 weeks (*P* < 0.05). Interestingly, fish-fed ginger nanoparticles showed non-significant differences from the control after 2 and 4 weeks (*P* > 0.05).

## Discussion

Water contamination with herbicides derivatives is a significant concern that threatens the viability and quality of the ecosystem and aquatic animals (Blahova et al. 2020; Bojarski and Witeska 2020; Stara et al. 2021). The integrated agriculture–aquaculture systems are a direct reason for pollution with herbicides that can interrupt the health of aquatic animals (Soror et al. 2021; Yousefi et al. 2021). This study showed the negative impacts of waterborne glyphosate and ginger nanoparticles' protective roles. The results indicated severe impacts of glyphosate toxicity on the hepato-renal function, stress-related biomarkers, and immune and antioxidative responses of Nile tilapia. Interestingly, dietary ginger and ginger nanoparticles ameliorated the impacts of glyphosate on Nile tilapia health.

This study showed that negative waterborne herbicides impacts are involved in the impairment of aquatic animals' metabolic and biochemical functions (Abdel-Warith et al. 2021; Samanta et al. 2014). Blood biochemical metabolites associated with liver (ALT and AST) and kidney functions (urea and creatinine) as well as the stress-related markers (cortisol and glucose) are usually detected in the blood to understand the impact of herbicides on fish health (Bacchetta et al. 2014; Ramaiah 2007). Besides, the level of blood proteins (total protein, albumin, and globulin) is influenced by the nutrient metabolism, hormones, enzymes, antibodies, and general immunity of fish, which can be disrupted by herbicide toxicity (Al-Ghanim et al. 2020; Gholami-Seyedkolaei et al. 2013). The results showed that Nile tilapia exposed to glyphosate have high ALT, AST, urea, and creatinine levels in blood samples after 2 and 4 weeks. The results agree with Yousefi et al. (2021), who indicated high levels of ALT and AST in common carp (*Cyprinus carpio*) exposed to glyphosate. Further, Dawood et al. (2020b) claimed high urea and creatinine levels in tilapia exposed to deltamethrin. The rise in ALT and AST was most likely due to cytolysis and enzyme leakage into the bloodstream, suggesting liver and kidney injury (Bacchetta et al. 2014). While increased urea level is associated with gills damage and high creatinine is associated with muscular dysfunction (Soror et al. 2021). The levels of blood proteins, albumin, and globulin were declined by glyphosate toxicity after 2 and 4 weeks, referring to reducing total protein resulting from liver tissue damage due to oxidative stress (Brum et al. 2018). In addition, the study showed high glucose and cortisol levels in the blood of tilapia exposed to glyphosate after 2 and 4 weeks. The results are similar to Yousefi et al. (2021), who reported high cortisol and glucose levels in common carp intoxicated with glyphosate. The cortisol hormone, which manages the organism's reaction to stresses, regulates the blood glucose level (Polakof et al. 2012). Cortisol is a sign of primary stress response, while an increase in glucose levels in serum indicates a secondary stress response in case of glyphosate toxicity (Langiano and Martinez 2008). The increased cortisol levels indicate that glyphosate generated stress in Nile tilapia which can be related to the high content of surfactants like polyoxyethylene amine (POEA) (Glusczak et al. 2007). Furthermore, glyphosate toxicity causes endocrine-disrupting effects by disrupting the function of the hypothalamus–pituitary–gonadal axis (Soso et al. 2007).

The disruption of ALT, AST, urea, and creatinine can also be related to increased glucose and cortisol levels (Soror et al. 2021; Yousefi et al. 2021). On the other hand, dietary ginger or ginger nanoparticles regulated the levels of ALT, AST, urea, creatinine, cortisol, and glucose, indicating healthy fish status. In a similar sense, Soror et al. (2021) reported that dietary ginger regulated the levels of ALT, AST, urea, creatinine, cortisol, and glucose and increased blood total protein, albumin, and globulin in Nile tilapia exposed to dimethoate. The reduction of ALT, AST, urea, and creatinine and the increased total protein, albumin, and globulin levels are probably attributed to ginger's antioxidative role, which balances the lipid peroxidation leading to inhibition of oxidative stress and thereby regular liver and kidney function (Ali et al. 2008).

The toxicity of glyphosate is responsible for generating reactive oxygen metabolites (ROS) that induce oxidative stress (Muhammad et al. 2021; Zheng et al. 2021). ROS production can interact with cellular lipid membranes leading to lipid peroxidation and damage to DNA and cellular function (Kavitha and Venkateswara Rao 2007). It has been reported that glyphosate could impair cellular function through cytoplasmic membrane toxicity and the production of oxidative stress (Yang et al. 2020; Yousefi et al. 2021). The concentration of malondialdehyde (MDA) is an indicator of lipid peroxidation (Zhang et al. 2020), while glutathione (GSH) is an antioxidant molecule (Forman et al. 2009), and an imbalance of ROS production and removal leads to the activation of GSH to counteract with high MDA concentration (Lackner 1998). This study detected the GSH activity and MDA levels in the gills and liver tissues. Gills in the first organ directly impacted by glyphosate toxicity result in the dysfunction of respiration and osmoregulation (Dawood et al. 2021). At the same time, liver tissue is responsible for detoxifying toxicants, xenobiotics, and secretion of pathogenic invaders (Tanaka et al. 1999). Thus, it is necessary to correlate the impact of glyphosate and the antioxidative capacity of gills and liver tissues that may explain the disrupted hepato-renal function and immune and antioxidative responses of Nile tilapia under the current trial conditions. The results showed high MDA levels and low GSH in fish exposed to glyphosate; however, dietary ginger regulated MDA and GSH after 2 and 4 weeks. Nile tilapia-fed dietary ginger or ginger nanoparticles displayed low MDA levels and high GSH, indicating high antioxidative capacity to cope with the impacts of glyphosate toxicity after 2 and 4 weeks. The results are in line with Yousefi et al. (2021) and Yang et al. (2019), who reported high MDA levels in common carp and Chinese mitten crab, respectively. Besides, Modesto and Martinez (2010), and Ma et al. (2019) reported a reduction in the antioxidative capacity of *Prochilodus lineatus* and common carp exposed to glyphosate. On the other hand, similar reports indicated that dietary ginger resulted in high antioxidative capacity in Nile tilapia (Soror et al. 2021) and common carp (Fazelan et al. 2020). Ginger contains polyphenols, gingerols, and shogaols with anti-inflammatory and antioxidative capacity, which can degenerate the ROS and protect the cell membrane from oxidation (Ali et al. 2008). In this context, dietary ginger enhanced the antioxidative capacity of Nile tilapia exposed to dimethoate (Soror et al. 2021).

There is a close connection between the antioxidative and immune responses, which can also be impaired by toxicity with glyphosate (Peillex and Pelletier 2020). In earlier reports, glyphosate toxicity resulted in immunosuppression of Nile tilapia (Zheng et al. 2021), Chinese mitten crab (Yang et al. 2019), and common carp (Yousefi et al. 2021), and silver catfish (Sutili et al. 2020). This study detected the lysozyme and total immunoglobulins after two and four weeks of glyphosate toxicity in Nile tilapia fed with or without ginger and its nanoparticles. Lysozyme activity and total immunoglobulins are innate immune responses that protect against infection with pathogenic bacteria through the damage of bacterial cell walls (Whyte 2007) and secretion of antibodies (Tellez-Bañuelos et al. 2010), respectively. The results showed impaired lysozyme and total immunoglobulin level in fish exposed to glyphosate, but fish treated with ginger had enhanced lysozyme activity and total immunoglobulin levels after 2 and 4 weeks. Yousefi et al. (2021) reported that common carp exposed to glyphosate had reduced lysozyme activity and total immunoglobulin levels. Herbicides toxicity induces immunotoxicity through a complex network of inflammatory cytokines release, immunoglobulins regulation, immune cell proliferation inhibition, and lysozyme activity changes (Yang et al. 2021). Exposure to glyphosate induces lipid peroxidation and oxidative stress causing deterioration of immune cells (B type) function. Thus, lowering the immunoglobulin production and inflammatory cytokines release and thereby immunosuppression (Chen et al. 2005; Mela et al. 2007). Interestingly, the inclusion of dietary ginger relieved the impacts of glyphosate and increased the lysozyme activity and total immunoglobulins under the current trial conditions. Similarly, the inclusion of ginger and its nanoparticles enhanced the lysozyme activity and total immunoglobulins in common carp (Fazelan et al. 2020; Mohammadi et al. 2020) and Nile tilapia (Brum et al. 2018). More research is needed to determine the specific mechanism of ginger's immunomodulatory effect. The presence of bioactive metabolites in ginger, including terpenes, zingiberol, zingiberene, zingiberene, zingerone, oleoresin, gingerol, shogaol, and paradol, is the most likely cause (Jesudoss et al. 2017). These components have an antibacterial and immunomodulatory role, which begins with the initiation of local intestinal immunity and, thereby, the entire body's immunity (Dawood 2021).

It is more likely that the nanoparticles of ginger are more effective than normal ginger particles in the protection against glyphosate-induced hepato-renal dysfunction, immunosuppression, and oxidative stress under the current trial conditions. Indeed, nano-engineered substances are illustrated as more effective and functional than the standard form (Korni and Khalil 2017). Nanoparticles have a small surface with high activity making them accessible during absorption and more functional inside the fish body (Singh et al. 2021). In this context, the inclusion of ginger nanoparticles is illustrated to be more effective than standard ginger form in Nile tilapia.

## Conclusion

In conclusion, glyphosate toxicity represents environmental agriculture- aquaculture risk resulting in the dysfunction of the hepato-renal tissues and impairment of the antioxidative and immune responses of Nile tilapia. However, dietary ginger and its nanoparticles markedly relived the toxic impacts of glyphosate, resulting in regulated hepato-renal tissues, antioxidative, and immune responses. Interestingly, ginger nanoparticles are superior to the standard ginger form in enhancing the antioxidative and immune responses of Nile tilapia exposed to glyphosate.

## Data Availability

Data and materials are available upon request.
